# Energy and nutrient intakes of Sri Lankan patients with type 2 diabetes mellitus: a cross-sectional survey

**DOI:** 10.1186/s13104-015-1732-5

**Published:** 2015-12-08

**Authors:** Arjuna Medagama, Devaka Fernando, Heshan Widanapathirana

**Affiliations:** Department of Medicine, University of Peradeniya, Peradeniya, Sri Lanka; Sherwood Forest Hospitals NHS Foundation Trust and Honorary Professor University of Kent Canterbury, Canterbury, UK

## Abstract

**Background:**

Sri Lanka has a high prevalence of type 2 diabetes mellitus. Energy and macronutrient intakes of diabetic patients have not been previously studied in this population. We aimed to clarify the energy and nutrient intakes among a group of type 2 diabetic patients attending a tertiary care diabetes facility in Sri Lanka.

**Methods:**

Nutritional and energy intake of 123 randomly selected patients with type 2 diabetes, aged 30–74 years was assessed using a 24-h dietary recall.

**Results:**

The mean energy intake for all participants was 1438 (SD 412) Kcal/day. The mean proportions of total carbohydrate, protein and fat comprising total energy intake were 68.1, 11.5 and 20.2 % respectively. The mean carbohydrate intake of 249.7 g/day comprised 50 % of rice. The mean daily protein, fat and dietary fibre intake was 42.5, 33 and 18.1 g respectively with a major contribution from plant sources. There was no significant difference in energy and nutrient intakes among the male and female participants.

**Conclusion:**

The present study provides the first pilot data on the energy and macronutrient intakes of diabetes patients in Sri Lanka. We clarified that these patients consumed an energy restricted, high-carbohydrate low fat diet compared to western diabetic patients. A larger nationwide dietary survey is recommended to confirm our findings.

## Background

Sri Lanka is a developing country with a population of 20 million and a heavy burden of diabetes.

Sri Lanka has a high burden of non-communicable diseases (NCD), and in 2005 the prevalence of hypertension, diabetes and dysglycaemia was 18.8, 13.8 and 14 % respectively from a survey performed in 3 of its 9 provinces [1]. A more recent nationwide survey found the prevalence of diabetes to be 10.3 % with a higher (21.8 %) prevalence of dysglycaemia. Prevalence of diabetes was notably higher among the urban population [2]. The mortality from cardiovascular disease in Sri Lanka is recognized to be one of the highest worldwide. While Sri Lanka recorded a mortality of 524 deaths from Cardiovascular and cerebro-vascular disease for 100,000 population, comparable figures for the united Kingdom, the United States of America and Australia were 427, 397 and 308 respectively [3]. This is probably contributed by unhealthy lifestyle, eating habits and the clustering of cardiovascular risk factors such as diabetes mellitus.

Rice is the principal staple of the Sri Lankan diet and other carbohydrates are consumed in lesser quantities. The high consumption of white rice is thought to increase the incidence and prevalence of diabetes [4]. A recent meta-analysis has shown an association between white rice consumption (with a high glycaemic index) and prevalence of diabetes in Asian populations [5].

A recent study by Jayawardena et al. found an average starch consumption of 14.1 servings per day among healthy Sri Lankan adults [6]. In this study, 70 % of the study population exceeded the recommended number of starch servings for a day for healthy adults. The added sugar use was very high, amounting to 3.56 servings per day and use of fresh fruits and vegetables was minimal. In another study, Jayawardena et al. established the mean energy intake of males and females to be 1913 kcal/day and 1514 kal/day respectively [7].

Energy and nutrient intakes of Sri Lankan patients with diabetes has not been studied before. This information is essential to gauge the effectiveness of current interventional strategies and for future planning.

The aim of this study was to calculate the mean daily energy and macronutrient intake of adult Sri Lankan type 2 diabetes patients, assess the socio-demographic correlates of energy intake and to find the proportion of macronutrient contribution to daily energy intake.

## Methods

This study was performed between May and August 2014, at the diabetes treatment facility at Teaching Hospital, Peradeniya, Sri Lanka. This facility has a patient base of 2200 registered patients and acts as a referral center for diabetes and caters to a muti-ethnic, multi cultural semi-urban community.

Over the 16-week study period, 8 random patient numbers were selected from each week giving a total of 128 participants. Five patients did not fulfill the inclusion criteria or declined to participate. Inclusion criteria were; adult patients with type 2 diabetes, having type 2 diabetes for longer than 6 months and having undergone nutrition counseling at the facility by a trained nurse in diabetes. Inability to give consent due to Physical, verbal or intellectual impairment was regarded as the exclusion criterion.

The selected participants were briefed and informed verbal consent was obtained and formally recorded. The ethical clearance for this study was obtained from the Ethical Review Committee of the Faculty of Medicine, University of Peradeniya Sri Lanka.

## Data collection

Two independent interviewers performed a 24-h dietary recall on the recruited patients. The data was recorded onto a pre-printed format, which allowed either weight of the portion or size of the portion using the methods described below, to be entered. The gathered data from the two interviewers were compared at the end of the day and if there was any discrepancy the patient was re-interviewed on another day. The participants were asked to recall what they ate during the preceding 24-h from midnight to midnight in chronological order. Participants were requested to report all foods and beverages consumed, excluding plain drinking water. If the participant was able to recall the foods for the preceding 24-h period the recall was considered reliable. Where the participant had difficulty in making a 24-hour recall the recall was considered unreliable and the data excluded from the study. The interviewers used common household utensils such as plates of varying sizes, bowls, cups, spoons and glasses to determine portion sizes. These were available in a variety of sizes for patients to go through and determine which best reflected their portion size. In instances that patients were able to recall weight of their portion, this was recorded. Common foods such as rice and curries were available within the interviewers room and the patients were asked to serve their usual portion size onto a plate and the weight of the portion was recorded. This was then compared with the portion size they had recalled using the common household utensils. If there was a discrepancy greater than 10 % between the recalled portion size and served portion size, the data was regarded as unreliable and discarded.

The use of a 24-h dietary recall is a potential source of bias as studies have shown that the validity of this tool differs among different subsets of a population [8].

## Calculation of energy and macronutrient intake

All the data recorded in the 24-h dietary recall was converted to grams and then the total intake of energy, carbohydrates, proteins, fat, sodium and dietary fibre were calculated. The United States Department of Agriculture (USDA) nutrient database USDA [9] was used to calculate the nutrient content of food in most instances and regional and locally accepted databases and texts [10] were used in cases of indigenous food items. In instances of mixed dishes they were disaggregated to the individual components and the weight of each constituent was determined by referring recipes and local housewives. Depending on the availability of data, either the raw or the cooked weight was used to determine the nutrient content of such mixed dishes. Raw ingredients were weighed to the nearest 1 g for the edible portions of the food.

The participants demographic details, the occupation, income, level of education, ethnicity, religion and the height and weight were also recorded according to standard methods.

## Statistical analysis

All statistical analyses were performed by using IBM SPSS statistics (Version 20). Descriptive data were presented as frequencies and percentages for categorical variables, and as mean ± standard deviation (SD) for continuous variables. Chi Square and independent *t* test was used to compare for differences and statistical significance level was set at P < 0.05.

## Results

### Socio-demographic profile

One hundred and twenty-three patients with type 2 diabetes were enrolled in the study following informed consent. All the patients were able to make a reliable recall of the foods consumed during the preceding 24-h. There were 43 (34.9 %) male and 80 (65.1 %) female participants. The mean age of the males and females were 57.8 and 55.2 years respectively. The majority of participants were older adults belonging to the age group of 50–59 years, were Singhalese and educated only up to secondary school level. 66 % of participants were overweight or obese when the proposed cut-off values for Asians [11] were used. The females had a higher mean BMI (25.2 kg/m^2^) compared to the males (24.39 kg/m^2^). The socio-demographic profile of the study population is shown in Table 1.

**Table 1:** Socio-demographic characteristics of study participants

Characteristics	Total (n = 123)			Men (n = 43)		Women (n = 80)		
	N	%		N	%	N		%
Age group (years)								
30–39	11	8.9		4	9.3	7		8.7
40–49	23	18.6		7	16.2	16		20
50–59	43	34.9		12	27.9	31		38.7
60–69	38	30.8		17	39.5	21		26.2
>70	8	6.5		3	6.9	5		6.25
Ethnicity								
Sinhala	109	88.6		39	90.7	70		87.5
Muslim	11	8.9		2	4.6	9		11.2
Tamil	3	2.4		2	4.6	1		1.25
Educational level								
No schooling	8	6.5		4	9.3	4		5
Up to 5 years	17	13.8		5	11.6	12		15
Up to O/L	70	56.9		26	60.4	44		55
Up to A/L	25	20.3		7	16.2	18		22.5
Graduate	3	2.4		1	2.3	2		2.5
BMI category								
≤22.9	36	33.96		16	42.1	20		29.41
23–24.9	21	19.81		7	18.42	14		23.33
25–27.5	24	22.64		7	18.42	17		25
>27.5	25	23.58		8	21.05	17		25

### Energy intake

The mean total daily energy intake of the participants was 1438.47 (SD 412) Kcal, with men (1477 kcal) consuming more energy than women (1417 kcal, P = 0.46) Younger patients with diabetes had significantly higher intake of energy (1783.18 kcal/day) over patients above the age of 70 years (1060.42 kcal/day), representing a gradual decline in energy intake with age. Energy intake was low (1339 kcal/day) in those with a normal BMI and high (1596 kcal/day) among those who were overweight. We did not observe a significant difference in energy intake according to ethnicity. Table 2 represents the distribution of energy intake of type 2 Sri Lankan adult diabetic patients.

**Table 2:** Energy intake (kcal) of participants by socio-demographic characteristics

	Mean	SD	Mean (males)	SD (males)	Mean (females)	SD (females)
Total sample	1438.47	498.9	1477.18	400.2	1417.65	402.4
Ethnicity						
Sinhala	1430.37	365.5	1477.4	382.6	1405.9	445.40
Muslim	1513.04	409.0	1545.3	544.2	1491.7	389.32
Tamil	1459.40	483.2	1403.8	357.2	1570.4	423.2
Age group						
30–39	1783.18	458.4	1669.98	139.5	1858.6	431.4
40–49	1504.50	547.8	1713.04	380.5	1413.2	505.5
50–59	1470.26	328.7	1458.93	297.1	1443.6	468.5
60–69	1343.89	576.6	1411.68	481.0	1334.7	361.7
>70	1060.42	523.9	1113.90	108.2	1028.3	450.9
Educational level						
No schooling	1183.4	498.5	1219.1	285.7	1147.6	356.1
Up to grade 5	1306.9	361.1	1340.3	393.0	1292.1	479.4
Up to O/L	1518.6	558.6	1577.3	412.5	1499.8	423.9
Up to A/L	1398.6	362.9	1381.2	453.2	1365.9	605.3
Graduate	1333.0	460.2	1258.1	345.9	1370.0	376.9
BMI category						
≤22.9	1394.2	463.5	1395.5	583.4	1351.5	396.1
23–27.5	1596.0	467.9	1696.1	411.1	1554.8	453.3
>27.5	1424.9	387.2	1560.2	339.4	1361.2	468.0

### Carbohydrate intake

The mean daily carbohydrate intake was 249.7 g without a significant difference between males and females. Carbohydrate intake amounted to 68.1 % of the total daily energy intake. Fifty percent of carbohydrate was consumed as rice, while pulses, vegetables, wheat-based products and fruits contributed 44.7 %. The daily carbohydrate intake decreased with age. The distribution of carbohydrate intake is given in Table 3.

**Table 3:** Carbohydrate, protein and fat intake of participants according to age and relative contribution to daily energy intake

Age category	Carbohydrate intake			Protein intake			Fat intake		
	Mean (g)	SD	% Energy from CHO	Mean (g)	SD	% Energy from protein	Mean (g)	SE	% Energy from fats
30–49	288.5	428.0	69	50.6	15.3	12.34	35.8	11.7	19.30
50–69	244.7	386.5	68	40.4	17.7	11.52	32.6	12.6	20.51
>70	174.8	334.8	65	36.2	12.8	12.63	28.0	14.7	21.65
All participants	249.7	412.0	68	42.5	16.0	11.5	33.0	11.5	20.5

### Protein intake

Sri Lankan adult type 2 diabetes patients recorded a mean daily protein intake of 42.53 g, without a significant difference between males and females. Animal proteins contributed to 36 % of the total protein intake and plant proteins 63.9 %. The protein intake in young patients (30–39 years) was significantly higher (52.4 g) compared to those over 70 years (34 g, P < 0.001). Patients with BMI over 27.5 consumed higher protein content (45.8 g) compared to those with BMI < 22.9 (37.7 g). The distribution of protein intake is illustrated in Table 3.

### Fat intake

The estimated mean daily fat intake was 33.05 g. The fat intake also decreased with age from 38.3 g in the 30–39 year age group to 27.2 g in the over 70-year age group. There was no difference in fat intake among obese, over-weight and normal categories of BMI. Animal fats contributed only 16.3 % to the total fat intake and plants 83.6 %. Subtypes of fats are not analyzed in this study.

### Dietary fiber

The mean daily dietary fiber intake was 18.13 g/day. The dietary fiber intake of 23.26 g/day among Tamil participants was higher than that of others.

The mean proportions of total carbohydrates, proteins and fats comprising total energy intake were 68.1, 11.5 and 20.2 % respectively. The percentage of participants falling within the recommended proportions for Carbohydrates, proteins and fat intake is given in Table 4.

**Table 4:** Percentages of energy contribution from carbohydrates, fat, protein recommendations and their relationship to recommended intakes

	Below recommendations (%)	Within recommendations (%)	Above recommendations (%)
Carbohydrates	*<45*	*45–65*	*>65*
	15.44 %	34.95 %	49.59 %
Fat	*<30*	*30–35*	*>35*
	97.56 %	0.81 %	1.62 %
Protein	*<10*	*10–20*	*>20*
	40.65 %	53.65 %	5.69 %

The values in italics represent the limits that are within current recommendations, below the recommendations and exceeding the recommendations when each nutrient intake is calculated as a percentage of the total daily energy intake

## Discussion

This is the first study performed among Sri Lankan type 2 diabetes patients to assess their energy and nutrient intake. We clarified that patients attending this multi-ethnic diabetes facility consumed a “high carbohydrate, low fat diet” compared to western patients. A high carbohydrate diet is considered to be one, which supplies more than 65 % of the total energy content from carbohydrates [12]. Our participants derived 68.1 % of their total energy intake from carbohydrates followed by 20.2 % from fats and 11.5 % from proteins.

Jayawardena et al. established the daily energy intake of healthy adult Sri Lankans to be 1912 kcal/day. In the same study the mean daily intake of carbohydrates, protein and fats were 304.4, 44.6 g and 35 g respectively. We note a considerable difference in the total energy (1912 vs. 1438 kcal) and carbohydrate (304.4 vs. 249.7 g) intakes among healthy Sri Lankans and type 2 diabetes patients. There was no difference in the protein (44.6 vs. 42.5 g) and fat intakes (35 vs. 33 g) between healthy and diabetic Sri Lankan adults. The method used for estimation of food intake in this study was comparable to ours. The same study as well as studies performed elsewhere noted a higher energy and carbohydrate intake among males compared to females. However, this study failed to demonstrate a significant variation in energy and carbohydrate intake between these 2 groups. This may be the result of dietary counseling, in which patients have been advocated a set portion size of their staple irrespective of their gender. The energy intake was highest among the patients belonging to the 30–39 year age group amounting to 1783.1 kcal/day. Energy intake was also high (1596 kcal/day) in the overweight group and declined in the obese group, probably a reflection of the more responsible eating habits of such patients. Two-thirds of the diabetic participants were overweight or obese as opposed to 34 % among healthy Sri Lankan adults [11] thereby making the reduced energy intake even more significant.

A significant reduction was observed in the energy, carbohydrate, protein and fat intakes with advancing age. This may reflect the general decrease of food consumption secondary to decreased appetite. This reduction was prominent in individuals over the age of 70 years. However, in view of the limited number of participants at the extremes of age in this study, the generalizability of the findings to the Sri Lankan diabetic population is limited.

We did not observe a significant difference in energy intake across different ethnic groups. However, such a difference would not be apparent considering the small sample size. Energy intake varied according to the level of education, with the least intake being (1183.7 kcal) in those without formal education to highest (1518.4 kcal) in those who have completed primary education only. It then declined in those who have completed secondary and tertiary education, probably reflecting more responsible eating habits with the advancing socio-economic status.

Other studies have found widely distributed energy intakes among diabetic patients ranging from 1384 to 1971 kcal/day [13]. The least was recorded among women participating in the National Health and Nutrition Examination Survey (NHANES) in the US and highest among South African men with type 2 diabetes. Horikawa et al. studying the energy intake of Japanese type 2 patients with diabetes found the mean daily energy intake to be 1737 kcal (SD 412) Kcal/day. This population is probably the closest in comparison to Sri Lankan eating habits.

Compared to these values, Sri Lankan diabetic patients seem to consume energy restricted diet. Unfortunately, there are no other studies, which quantified the energy, and macronutrient intakes of Sri Lankan diabetic patients for comparison.

This study only included type 2 diabetic patients. A previous study among healthy Sri Lankan adults noted a total daily energy intake of 1912 kcal/day [7]. Compared to this study, we noted a difference in energy intake among the participants of our study. A decline in energy intake in diabetic patients compared to healthy free-living subjects has been noted elsewhere as well. In the United States, the energy intake decreased from 2591 to 1612 kcal/day in healthy and diabetic populations respectively [14, 15].

Western guidelines (American Association of clinical Endocrinologists [16], European Association for the Study of Diabetes [17], Canadian Diabetes Association [18] recommend carbohydrates to contribute 45–65 %, fat 30–35 %, and protein 10–20 % of the total daily energy intake. Thirty-five percent of our study population fulfilled the recommendation for carbohydrate intake, while 50 % exceeded it. On the other hand, the protein and fat intakes were within the recommendations. Proportion of energy intake from carbohydrate is generally low among western diabetic patients and high among those of Asian origin where rice is the staple. National Health and Nutrition Examination survey of the US, Diabetic Educational Eating Plan Study and the Strong Heart Study among American Indians found that carbohydrates contributed 48–49 % to the daily energy intake [15, 19]. On the other hand Asian diabetic populations generally derive a larger proportion of the total energy from carbohydrates. Lee et al. [20] found that carbohydrates contributed 66–68 % towards total energy intake in Koreans. In our study although the total energy intake is reduced, a large proportion (68.1 %) is dependent on carbohydrates.

According to prevailing guidelines fats should contribute no more than 30–35 % of the daily energy intake. Studies performed on US diabetic populations show fats contribute 33–44.6 % [15, 19]. Asian diabetic populations on the other hand have a reduced fat intake and generally contributes to only 16–28 % [13, 20]. In our study the mean fat intake was 33 g and contributed only 20.2 % to the energy intake. This is similar to healthy Sri Lankans who consumed 35 g of fats per day [7]. Fat of plant origin was the major contributor (83.6 %) towards the total daily fat intake in our study, while animal fats contributed only 16.3 %.

Coconut milk and raw grated coconut with added condiments (*Sambol*) is a traditional constituent of Sri Lankan recipes and usually contributes substantially to the fat intake. However, fat intake remains largely unchanged between healthy and diabetic Sri Lankan adults.

Healthy American men and women consume around 97 and 65 g of protein each day [14] and healthy Sri Lankans around 44.6 g [7]. We observed a daily protein intake of 44.4 and 41.5 g in Sri Lankan male and female diabetic patients, contributing to 11.5 % of the total energy intake. The highest protein intake of 47.8 g was noted in the overweight category reflecting the higher energy intake noted in this group earlier. The least intake of 34 g was noted in the over 70-year age group reflecting the absolute reduction of food intake in this group.

In our study, animal proteins contributed only 36.06 % to the total protein intake in contrast to the US population where 69 % of the content was animal in origin [14]. Higher contribution of plant protein to Sri Lankan diet is previously documented [7].

Increased dietary fiber consumption is encouraged in type 2 diabetes as it improves fasting plasma glucose (FPG) and HbA1C [12]. The current recommendation for dietary fibre is to consume 14 g for every 1000 kcal for healthy individuals [7]. The mean dietary fiber intake of 18.13 g in our study largely met these recommendations. Similar values (11.4–20.5 g/day) have been observed in other studies in western and Asian diabetic populations [13].

The mean BMI of the males and females in our study was 24.39 and 25.2 kg/m^2^ respectively. In western populations where the energy intake ranged from 1384 to 1852 kcal/day, the corresponding BMI also was higher, ranging from 30.5 to 32.8 [15].

### Strengths and limitations

The data provides a general overview of the energy and macronutrient intakes of a Sri Lankan semi-urban diabetic population, where previous data was unavailable. We acknowledge the following limitations of the study. The sample size is small and therefore the ethnic and socio-economic groups may be underrepresented. There is limited generalizability of the data due to the sample being drawn from a predominantly semi-urban population. The use of a single 24-h dietary recall may have introduced a potential recall bias.

## Conclusion

We clarified that diabetic patients attending our treatment facility consumed an energy restricted “high carbohydrate low fat diet” and that their energy intake was low, compared to other Asian and Western diabetic populations. Protein and fat intake was predominantly of plant origin. A larger nation-wide survey is recommended.
